# Umbilical Cord-Derived Wharton’s Jelly for Regenerative Medicine Applications: A Systematic Review

**DOI:** 10.3390/ph14111090

**Published:** 2021-10-27

**Authors:** Benjamin J. Main, Nicola Maffulli, Josiah A. Valk, Hugo C. Rodriguez, Manu Gupta, Saadiq F. El-Amin, Ashim Gupta

**Affiliations:** 1 Department of Orthopaedic Surgery, Beaumont Hospital Farmington Hills, Farmington Hills, MI 48336, USA; benjamin.main@beaumont.org (B.J.M.); Josiah.valk@beaumont.org (J.A.V.); 2 Department of Musculoskeletal Disorders, School of Medicine and Surgery, University of Salerno, 84081 Baronissi, Italy; n.maffulli@qmul.ac.uk; 3 Centre for Sports and Exercise Medicine, Barts and the London School of Medicine and Dentistry, Queen Mary University of London, Mile End Hospital, London E1 4DG, UK; 4 School of Pharmacy and Bioengineering, Faculty of Medicine, Keele University, Thornburrow Drive, Stoke-on-Trent ST4 7QB, UK; 5 Holly Cross Orthopedic Institute, Fort Lauderdale, FL 33334, USA; hcrodrig2112@gmail.com; 6 Polar Aesthetics Dental & Cosmetic Centre, Noida 201301, Uttar Pradesh, India; manu6771@yahoo.co.in; 7 El-Amin Orthopaedic and Sports Medicine Institute, Lawrenceville, GA 30043, USA; dr.saadiqelamin@gmail.com; 8 BioIntegrate, Lawrenceville, GA 30043, USA; 9 Future Biologics, Lawrenceville, GA 30043, USA

**Keywords:** umbilical cord, Wharton’s jelly, regenerative medicine, mesenchymal stem cells, mesenchymal stromal cells, Wharton’s jelly mesenchymal stem cells, musculoskeletal injuries, osteoarthritis, PRISMA, systematic review

## Abstract

Musculoskeletal ailments affect millions of people around the world and place a high burden on healthcare. Traditional treatment modalities are limited and do not address underlying pathologies. Mesenchymal stem cells (MSCs) have emerged as an exciting therapeutic alternative and Wharton’s jelly-derived mesenchymal stem cells (WJSCs) are some of these. This review reports the clinical and functional outcomes of the applications of WJSCs in orthopedic surgery. A systematic review was conducted utilizing the Preferred Reporting Items for Systematic Reviews and Meta-analyses (PRISMA) guidelines. The studies that used culture-expanded, mesenchymal stem or stromal cells, MSCs and/or connective tissues procured from Wharton’s jelly (WJ), from January 2010 to October 2021, were included. Conventional non-operative therapies and placebos were used as comparisons. Six studies that directly discussed WJSCs use in an animal model or the basic scientific testing using an injury model were identified. Five publications studied cartilage injury, three studied degenerative disc disease, one was related to osteoarthritis, and one was related to osteochondral defects. The results of these studies suggested the benefits of WJSCs in the management of these orthopedic pathologies. To adequately assess the safety and efficacy of WJSCs in orthopedic surgery, further randomized controlled clinical studies are necessary.

## 1. Introduction

Orthopedic musculoskeletal ailments involve inflammatory and/or degenerative conditions in muscles, tendons, ligaments, nerves, and bones. These conditions are estimated to affect one in four people in developed countries, thus representing a significant burden on healthcare [1]. Traditionally, musculoskeletal injuries are handled with activity modification, physical therapy, immobilization, pharmacological drugs, and surgical management once conservative treatments are exhausted. These treatment modalities are imperfect, often attempting to limit pain instead of focusing on the underlying pathology [2,3].

The field of regenerative medicine has undergone a tremendous growth as of late, especially the field of orthopedic surgery [4]. Mesenchymal stem cells (MSCs) offer regenerative potential, aiming to slow or halt chronic disease as well as improve function and patient satisfaction [5,6,7]. MSCs can be harvested from autologous bone marrow concentrate (BMC), adipose tissue, and allogenic umbilical cord-derived Wharton’s jelly (UC derived-WJ) [8,9,10,11]. Given the increased patient awareness and recent advances in MSC therapy, these biologic approaches are becoming more common in orthopedic practice [4].

BMC and adipose-derived stem cells (ADSCs) are clinically available and have a long history of being used with robust clinical data, in comparison to other sources [12]. However, both stem cell sources pose limitations. BMC is associated with surgical site morbidity from the aspiration procedure, a limited number of MSCs within the aspirated bone marrow concentrate, and signs of early senescence [13]. Adipose-derived stem cells exhibit promising short-term clinical results, but research on this is minimal with limited randomized controlled trials and a lack of adequate long-term follow-up. Adipose-derived stem cells are also associated with donor site morbidity from the extraction procedure [14].

WJ is an allogenic tissue comprised of connective tissue located within the umbilical cord. Wharton’s jelly resists torsional and compressive stresses during fetal development levied upon the umbilical vessels. The primitive mesenchymal stem cells reside within the UC-derived WJ [15]. These perinatal MSCs resemble embryonic stem cells (ESCs) but exhibit many properties of adult MSCs. Wharton’s jelly-derived mesenchymal stem cells (WJSCs) exhibit lower expression levels of pluripotent markers compared to ESCs, indicating multipotency rather than pluripotency [16,17]. Wharton’s jelly contains the highest concentration of MSCs/mL compared to other tissue types. UC-derived Wharton’s jelly also exhibits rich extracellular matrix (ECM) components such as collagen, hyaluronic acid, chondroitin sulfate, and sulfated proteoglycans [18,19].

Wharton’s jelly is easily accessible and available in comparison to autogenic tissues. The UC, and the Wharton’s jelly within it, is an after-birth tissue, and is normally discarded after every birth, presenting ample opportunity for harvest [20]. The ease of collection offers several benefits over the existing BMSC and ADSC harvest, both of which may present donor site morbidity. This factor, in addition to the multipotency of WJSCs, makes Wharton’s jelly a likely source of MSCs for regenerative medicine applications in the field of orthopedic surgery [21].

Given the possible advantages of Wharton’s jelly, the present systematic review evaluated the quality of the published evidence related to the safety and efficacy of WJSCs for orthopedic regenerative applications. The primary goal of this review is to document the clinical and functional outcomes of WJSCs for orthopedic, regenerative medicine applications. The secondary goal of this review is to identify the methodological characteristics associated with the application outcomes.

## 2. Results

**Figure 1:**
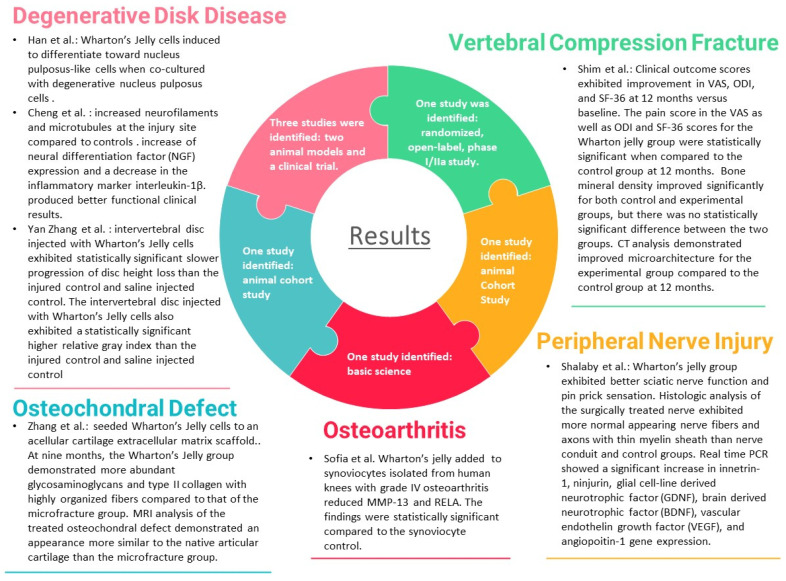
Summary of results of articles that met search criteria.

Of the 20 publications describing the use of Wharton’s jelly in regenerative medicine application for orthopedic surgery, only seven directly discussed human WJSCs are undergoing human or animal model testing, or basic scientific testing using an injury model. Five of the publications studied cartilage injury: three related to degenerative disc disease, one related to osteoporotic vertebral compression fractures, one related to osteoarthritis, and one related to osteochondral defect. Figure 1 and Table 1 summarizes these seven articles.

### 2.1. Degenerative Disc Disease

Han et al. analyzed the effect of Wharton’s jelly cells on degenerative nucleus pulposus cells isolated from a degenerative intervertebral disc. Wharton’s jelly cells were co-cultured in vitro with nucleus pulposus cells for seven days with and without direct cell-to-cell contact. Gene expression was quantified using a polymerase chain reaction (PCR) analysis. Compared to a Wharton’s jelly cell control and a degenerative nucleus pulposus cell control, the expression of type II collagen, aggrecan, and SOX-9 were significantly elevated for Wharton’s jelly and the nucleus pulposus co-culture. The gene expression was at its highest with direct cell-to-cell contact using a ratio of 75:25 Wharton’s jelly cells to nucleus pulposus cells. The polymerase chain reaction gene expression of the co-cultured Wharton’s jelly cells and degenerative nucleus pulposus cells differed from each individual control. Human Wharton’s jelly cells could be induced to differentiate toward nucleus pulposus-like cells when co-cultured with degenerative nucleus pulposus cells [22].

Cheng et al. analyzed the effects of a single injection of Wharton’s jelly-derived cells on acute spinal cord injury in a rat model. At day 28, the L3 transected rats that received the Wharton’s jelly injection exhibited a statistically significant increase in motor function versus the rats that did not receive Wharton’s jelly. The use of transmission electron microscopy in the Wharton’s jelly group demonstrated considerably increased neurofilaments and microtubules at the injury site compared to the rats that did not receive Wharton’s jelly. Additionally, the authors demonstrated an increase in the neural differentiation factor (NGF) expression and a decrease in the inflammatory marker interleukin-1β. Wharton’s jelly cells injected after spinal cord injury in a rat model produced better functional clinical results [23].

Yan Zhang et al. studied the effects of Wharton’s jelly on degenerated nucleus pulposus in a canine model. The degeneration of L4-5, L5-6, and L6-7 were induced. Intervertebral discs were exposed through an anterolateral approach, and 14.5 ± 2.7 mg of nucleus pulposus was aspirated from each disc. Four weeks after the procedure, 10^6^ Wharton’s jelly cells labeled with a viral vector were injected into L6-7. L5-6 was injected with saline, L4-5 served as the injured control, and L3-4 served as the uninjured control. Throughout the experiment, the disc height index and relative gray index were measured via a radiograph and magnetic resonance imaging (MRI), respectively. At 24 weeks, the intervertebral disc injected with Wharton’s jelly cells exhibited a statistically significant slower progression of disc height loss than the injured control and saline injected control. The intervertebral disc injected with Wharton’s jelly cells also exhibited a statistically significant and higher relative gray index than the injured control and saline injected control. At 20 weeks after injection, the discs were removed. Immunohistochemistry confirmed the presence of Wharton’s jelly cells at 20 weeks [24].

### 2.2. Osteoporotic Vertebral Compression Fracture

Shim et al. presented the results of a randomized, open-label, phase I/IIa study examining the safety and effectiveness of managing osteoporotic vertebral compression fractures with WJSCs and teriparatide. Twenty subjects were followed for 12 months. All subjects received a daily subcutaneous injection of 20 mg teriparatide and 20 mg oral bazedoxifene daily for 6 months. The subjects in the experimental group underwent an injection of WJSCs intramedullarily on day 0 and intraveniously on day 7. Three subjects from the control group dropped out because of an adverse reaction to teriparatide. Four subjects in the experimental group experienced an adverse event. Three patients chose to drop out of the study: one secondary to a urinary tract infection shortly after WJSC injection, another secondary to a pulmonary embolus discovered 30 days after WJSC injection on chest CT, and the third secondary to a diagnosis of pancreatic cancer discovered on CT. The clinical outcome scores exhibited statistically significant improvements in VAS, ODI, and SF-36 after 12 months versus the baseline. The pain score in the VAS, as well as the ODI and SF-36 scores, for the experimental group were statistically significant when compared to the control group at 12 months. Bone turnover markers measured did not demonstrate a statistical significance between the control and experimental groups. Bone mineral density improved significantly for both the control and experimental groups, but there was no statistically significant difference between the two groups. CT analysis demonstrated an improved microarchitecture for the experimental group compared to the control group at 12 months [25].

### 2.3. Peripheral Nerve Injury

Shalaby et al. examined the effect of Wharton’s jelly cells added to a nerve conduit on the functional recovery of a 10 mm sciatic nerve deficit. At 12 weeks, the Functional Recovery Index was −5.2 ± 2.1 in the uninjured control group, −55.3 ± 12.3 in the injured control group, −23.8 ± 5.6 in the injured group treated with nerve conduit alone, and −9.8 ± 2.5 in the injured group treated with nerve conduit and Wharton’s jelly cells. There was a greater significant improvement in the Wharton’s jelly group. For the pin prick-functional analysis, there was a statistically significant improvement in the treated groups, but no significance for the nerve conduit group and the group treated with Wharton’s jelly. Histologic analysis of the surgically treated nerve exhibited more normally appearing nerve fibers and axons with thin a myelin sheath than nerve conduit and control groups. The real-time PCR showed a significant increase in innetrin-1, ninjurin, the glial cell-line-derived neurotrophic factor (GDNF), the brain-derived neurotrophic factor (BDNF), the vascular endothelin growth factor (VEGF), and angiopoitin-1 gene expression versus the other three groups [26].

### 2.4. Osteoarthritis

Sofia et al. conducted a basic science study observing the matrix metalloproteinase-13 (MMP-13) gene expression of synoviocytes isolated prior to a total knee arthroplasty versus those cells combined with Wharton’s jelly cells. This study analyzed the gene expression of two pro-inflammatory markers: MMP-13 and RELA. The addition of Wharton’s jelly to synoviocytes isolated from human knees with grade IV osteoarthritis reduced the expression of MMP-13 and RELA. The findings were statistically significant compared to the synoviocyte control [27].

### 2.5. Osteochondral Defect

Zhang and colleagues seeded Wharton’s jelly cells to an acellular cartilage extracellular matrix scaffold. The seeded scaffold was then tested against a microfracture for the restoration of a 6.5 mm diameter, femoral condyle osteochondral defect in a caprine model. At nine months, the Wharton’s jelly group demonstrated more abundant glycosaminoglycans and type II collagen with highly organized fibers compared to that of the microfracture group. The modulus of elasticity was 2.9 ± 9 MPa for the Wharton’s jelly group compared to 2.2 ± 5 MPa for the microfracture group. An MRI analysis of the treated osteochondral defect demonstrated an appearance that was similar to the native articular cartilage than the microfracture group. Of note, two knees of goats from the microfracture group were deemed to have a meniscus tear at the time of euthanasia [28].

## 3. Discussion

The Wharton’s jelly extracellular matrix is partly comprised of glycosaminoglycans and collagen, similar to cartilage [29,30,31]. This relationship makes Wharton’s jelly cells an excellent source for cartilage tissue engineering [32,33,34,35]. Chondrocytes and human Wharton’s jelly cells also express aggrecan, type II collagen, and hyaluronic acid [29]. These similarities in the relationship and property between chondrocytes and Wharton’s jelly, as well as their regenerative ability, make WJSCs an excellent source for cartilage regeneration purposes.

The present study evaluated the quality of published evidence regarding the safety and efficacy of WJSCs for orthopedic regenerative medicine applications. To date, there is only one scientific publication in the literature using culture-expanded WJSCs for orthopedic applications in clinical practice. Shim et al. presented the results from a randomized, open-label, phase I/IIa study examining the safety and effectiveness of managing osteoporotic vertebral compression fractures with WJSCs and teriparatide. The study reported a statistically significant improvement in the pain and functional scores for the experimental group compared to the control. The bone mineral density and bone turnover markers did not significantly differ from the control and experimental subjects. Of note, that study included a large percentage of participants who did not complete the 12-month trial. More clinical research must be completed to determine the safety and efficacy of WJSCs in a human compression fracture model.

There is another current phase I/II clinical trial at the Medical University of Warsaw evaluating the efficacy of an intra-articular injection of 10^7^ WJSCs for moderate hip, knee or glenohumeral osteoarthritis. One hundred subjects will receive an injection of WJSCs every three months for one year. The subjects will be followed throughout the study and 24 months post-administration of last injection, studying clinical responses, inflammatory markers, and magnetic resonance imaging. The clinical trial is still in the recruitment phase [36].

Aging negatively affects stem cells. This makes stem cells cultured from the umbilical cord or placenta advantageous over stem cells cultured from adipose tissue, bone marrow, or other autogenic adult cell sources. Birth-derived products have shown potential for use in the orthopedic sector. Multiple companies now have a flowable placental allograft formulation that is under consideration for approval by the US FDA as Human Cells, Tissues, and Cellular and Tissue-Based Products (HCT/P’s) for eventual clinical use. While there is growing interest from companies, consumers, and healthcare providers, a lack of insurance reimbursement and limited safety and efficacy studies limit the development and use of these products. Additionally, to our knowledge the current commercial products on the market do not offer living cells [37].

Similar to placental tissue, Wharton’s jelly is obtained after birth. This alleviates the controversial aspects of harvesting embryonic cells. Unique to WJSCs, the process used to extract WJSCs can be performed without the use of digestive enzymes, cryoprotectants, or the in vitro expansion of cells [1]. All reviewed publications showed a certain degree of effectiveness in handling orthopedic injuries when compared with the controls. Only one study included a placebo control, and none of the studies compared the effectiveness of WJSCs to different stem cell types. Of the three studies related to intervertebral disc injury, one of the studies examined peripheral nerve injury, and the remaining two studies focused on osteochondral injury. Given the limited number of published preclinical studies, the variability between the animal models used, the specific injury model, and route of administration, it was not feasible to perform a comparative analysis. However, another review may be completed in the near future with an emphasis on preclinical models as the data seem to be positive in several studies [38,39].

In the study by Yan Zhang et al., human Wharton’s jelly-derived cells were injected into the intervertebral disc in a canine model. Immunohistochemistry confirmed the presence of Wharton’s jelly cells at 20 weeks [24]. Similar results were seen in a study by Y Zhang, with Wharton’s jelly cells seeded on an acellular collagen extracellular matrix scaffold, which maintained the presence of the Wharton’s jelly cells without an excess immune attack for 14 days in synovial fluid [28]. These results suggest that Wharton’s jelly cells are immunologically privileged and have the potential to be used for allogenic transplantation. A previous study in a rabbit model demonstrated that most human Wharton’s jelly cells did not express HLA-DR (MHC-II), suggesting Wharton’s jelly cells to be hypoimmunogenic [40]. Furthermore, studies demonstrate the anti-inflammatory and immunomodulatory effects of WJSCs [41,42].

## 4. Materials and Methods

**Figure 2:**
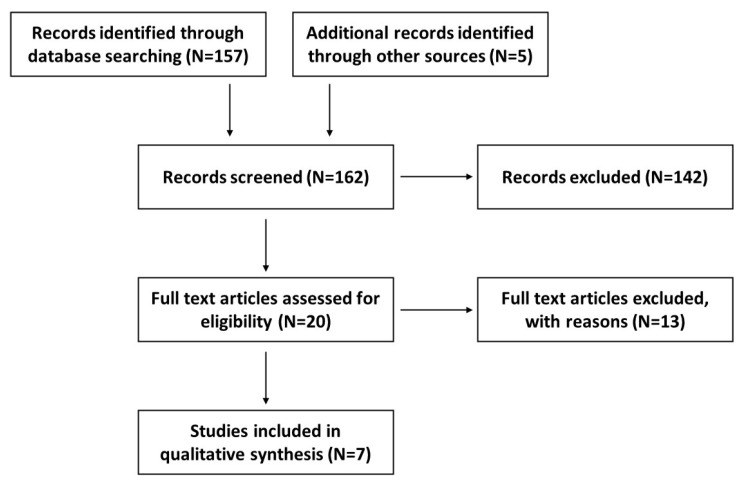
A flow diagram outlining the record identification and selection process.

The methodology of this systematic review followed the protocol that was previously published and registered on the international prospective register of systematic reviews (PROSPERO), registration number CRD42020182487 [43]. The various steps described in our published systematic review protocol were fully followed [41]; however, the search time range for published data was broadened from January 2010 to October 2021. A flow diagram (Figure 2) shows the record selection process.

## 5. Conclusions

The literature describing the use of WJSCs for musculoskeletal regenerative medicine is limited. However, the safety profile and the effectiveness in managing musculoskeletal ailments described in this review are encouraging. The benefits of WJSCs for cartilage restoration seem to be the most promising, given the similarities between chondrocytes and Wharton’s jelly cells and the cellular matrix of cartilage and Wharton’s jelly. Further well-designed and appropriately powered, prospective, non-randomized and randomized studies evaluating the safety and efficacy of WJSCs in a human model are warranted to justify their clinical use.

## Figures and Tables

**Table 1:** Summary of articles that met selection criteria.

Authors	Design	Group Controls	Group Interventions	Outcome Measurement
Degenerative Disc Disease				
Han et al., 2018	Basic Science	Fluorescently labeled human Wharton’s jelly cells (10^6^)	+ Degenerative human nucleus pulposus cells with cell-to-cell contact for 7 days; + Degenerative human nucleus pulposus cells without cell-to-cell contact for 7 days;	PCR gene expression of MSC markers
Cheng et al., 2016	Animal Model: Cohort	Rat model with sham surgery.	+ Incomplete transection of spinal cord at L3 + 10^6^ Wharton’s jelly cells injected into the femoral vein following L3.	Motor recovery using BBB scale at time points up to 28 days; PCR; Histologic pathology at 28 days;
Yan Zhang et al., 2015	Animal Model: Cohort	Canine model with L3-4 as uninjured control and L4-5 as the degenerative control.	+ 10^6^ WJC labeled via viral vector to L6-7	Radiographs; MRI; Biomechanical testing at 24 weeks; PCR at 24 weeks; Histologic analysis at 24 weeks;
Osteoporotic Vertebral Compression Fracture				
Shim et al., 2021	Human Model: Phase I/IIa Randomized Control Trial	Postmenopausal 50–89 year old females with recent (<6 weeks) single-level compression fracture and a diagnosis of osteoporosis were given a subcutaneous injection of 20 mg of teriperatide and 20 mg oral bazedoxifene daily for 6 months.	+ 4 × 10^7^ WJSC injected intramedullary into fractured vertebrae at day 0 and 2 × 10^8^ WJSC injected intraveniously at day 7.	Clinical assessment (VAS, ODI, SF-36); Bone mineral density via DEXA scan; Bone turnover markers; Radiographical analysis;
Peripheral Nerve Injury				
Shalaby et al., 2017	Animal Model: Cohort	Rats without sciatic nerve injury.	+ Sciatic nerve 10 mm induced injury. + Sciatic nerve 10 mm induced injury with nerve conduit. Sciatic nerve 10 mm induced injury with nerve conduit housing Wharton’s jelly cells.	Characterization of Wharton’s jelly cells; Functional nerve analysis; Histologic analysis
Osteoarthritis				
Sofia et al., 2019	Basic Science	Synoviocytes isolated from synovial tissue removed during total knee arthroplasty.	+ Wharton’s Jelly cells for 24 h and 48 h.	PCR gene expression and concentration
Osteochondral Defect				
Y Zhang et al., 2018	Animal Model: Cohort	Caprine model with induced 6.5 mm diameter osteochondral defect	+ Microfracture. + Implantation of acellular cartilage extracellular matrix scaffold seeded with Wharton’s jelly cells.	Histologic analysis; Immunochemistry and immunofluorescence; Biomechanical testing; MRI evaluation

## Data Availability

The data are contained within the article.
